# Low-Cost Sensor for Lycopene Content Measurement in Tomato Based on Raspberry Pi 4

**DOI:** 10.3390/plants12142683

**Published:** 2023-07-18

**Authors:** Marcos-Jesús Villaseñor-Aguilar, José-Alfredo Padilla-Medina, Juan Prado-Olivarez, José-Erinque Botello-Álvarez, Micael-Gerardo Bravo-Sánchez, Alejandro-Israel Barranco-Gutiérrez

**Affiliations:** 1 Departamento de Ingeniería de Robótica y de Datos, Universidad Politécnica de Guanajuato, Cortazar 38496, Mexico; mvillasenor@upgto.edu.mx; 2 Tecnológico Nacional de México en Celaya, Celaya 38010, Mexico; alfredo.padilla@itcelaya.edu.mx (J.-A.P.-M.); juan.prado@itcelaya.edu.mx (J.P.-O.); enrique.botello@itcelaya.edu.mx (J.-E.B.-Á.); gerardo.bravo@itcelaya.edu.mx (M.-G.B.-S.)

**Keywords:** artificial neuronal network, fuzzy logic, HPLC, image, lycopene, Raspberry Pi 4, tomato

## Abstract

Measuring lycopene in tomatoes is fundamental to the agrifood industry because of its health benefits. It is one of the leading quality criteria for consuming this fruit. Traditionally, the amount determination of this carotenoid is performed using the high-performance liquid chromatography (HPLC) technique. This is a very reliable and accurate method, but it has several disadvantages, such as long analysis time, high cost, and destruction of the sample. In this sense, this work proposes a low-cost sensor that correlates the lycopene content in tomato with the color present in its epicarp. A Raspberry Pi 4 programmed with Python language was used to develop the lycopene prediction model. Various regression models were evaluated using neural networks, fuzzy logic, and linear regression. The best model was the fuzzy nonlinear regression as the RGB input, with a correlation of R^2^ = 0.99 and a mean error of 1.9 × 10^−5^. This work was able to demonstrate that it is possible to determine the lycopene content using a digital camera and a low-cost integrated system in a non-invasive way.

## 1. Introduction

Fresh tomatoes are the most consumed fruit worldwide for their nutritional value and health benefits [1]. In 2021, the United Nations Food and Agriculture Organization (FAO) reported that world tomato production was more than 189 million tons [2]. It should be noted that tomatoes are a nutritional source of potassium, vitamin E, A, C, phosphorus, and antioxidants. The intake of this vegetable prevents the development of lung, stomach, and prostate cancer and cardiovascular diseases [3].

Traditionally, the harvest moment of this fruit is terminated visually by a farmer, who identifies the different degrees of maturity [4]. Nevertheless, this process can be affected by lighting, environmental conditions, and a lack of harvester experience [5]. The tomato ripening process involves physiological and biochemical changes that include ethylene, carotenoids, and cell wall metabolism [6,7]. Lycopene is a linear carotenoid that synthesizes through a pathway starting from geranyl diphosphate [8]. It accounts for about 80–90% of the carotenoids found in tomatoes. This is a natural pigment that generates the red coloration, which is used as a quality parameter by consumers [9,10]. Furthermore, lycopene has a high economic impact on the development of products in the pharmaceutical industry, such as clinical products, shakes, vitamins, proteins, and sports supplements [11].

The conventional technique used in laboratories for the measurement of lycopene is high-performance liquid chromatography (HPLC). This is a technique for separating non-volatile or thermolabile species from a sample using chemical interactions between the analyte and the chromatographic column [12]. Measurements made with HPLC are represented by a chromatogram. The measurement process using HPLC is costly in terms of time and money. In addition, another drawback of HPLC is the invasion of the fruit to obtain a homogeneous sample that will facilitate the extraction process of the lycopene. This is perform ed with the use of highly toxic chemical solvents such as ethanol, acetone, petroleum ether, hexane, benzene, chloroform itself, or their combinations [13,14].

On the other hand, color measurement is one of the unconventional laboratory techniques that correlates with lycopene. The CIEL*a*b* color space model is the most widely used due to its ability to classify the stages of tomato maturity. This is used because the *a axis is sensitive to changes in color from red to green and, in the case of the *b axis, from yellow to blue. This carotenoid can be estimated using the color descriptors *a, *a/*b, (*a/*b)^2^, hue, and chroma. Regarding the ratio a*/b*, it is commonly used in the generation of mathematical models due to its low computational cost. It should be noted that the color measurement of the samples is carried out with laboratory equipment under controlled conditions, such as the Konica-Minolta CR200, CR400, CR410, and CM-2002. Another device used is the Ocean Optics STS-VIS detector, a spectrometer with a fiber optic input. This has been used to develop a portable LED-based colorimeter using the reflectance spectrum of tomato in the range of 400–750 nm, estimating lycopene concentrations of 70 and 550 mg × kg of fresh weight skin. Another widely used technique is Vis-NIR spectra; this technique presents slightly better results in predicting lycopene than using the ratio a*/b* [15,16,17,18,19].

It is necessary to highlight that the Raspberry-Pi-embedded system has become very important in scientific and engineering applications [20]. For example, it helps eliminate the subjectivity and complexity of the measurement systems of the physicochemical properties and quality parameters of fruits on a minicomputer design and camera [21,22,23,24,25,26]. The present work proposed a methodology for developing a non-invasive sensor for measuring tomato lycopene in situ. The sensor uses artificial vision and artificial intelligence techniques implemented on a Raspberry Pi 3. The device operates using various optical filters that improve the spectral selectivity of the images to be processed. Together, it has an innovative structure made by 3D printing that eliminates lighting variations.

This work is composed of four sections. Section 2 shows the results of the mathematical models based on artificial neuronal networks and fuzzy logic. Section 3 presents the results of the mathematical models based on artificial neuronal networks and fuzzy logic. Subsequently, Section 4 indicates the materials and methods used to design the sensor. This section considers how the samples were chosen, the analysis using HPLC, and the sensor configuration process. Section 5 presents the conclusions.

## 2. Results

**Figure 1:**
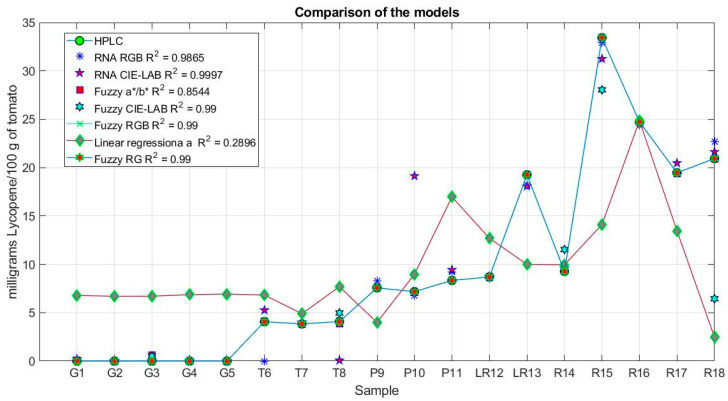
Predictions of the sensor models.

**Figure 2:**
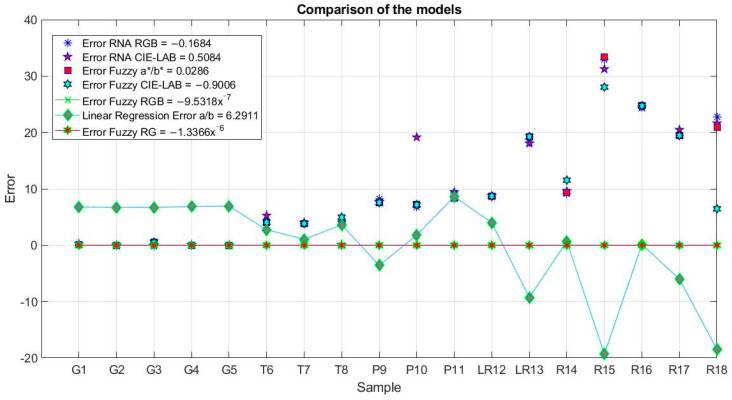
Average error of the predictions of sensor models.

Figure 1 and Figure 2 show the measurements and errors of the models proposed in the sensor. The measurements made with the HPLC for each sample can be seen in Figure 1. These correspond to eighteen circular green markers. It can be seen that the best model was Model 4, which used the RGB components as input, with a correlation of 0.99 and an average error of 1.9 × 10^−5^. Model 4 has a 99% coincidence with the measurements made by the HPLC. Models 5 and 6 presented slightly lower results with average errors of 9.3973 × 10^−5^ and −7.8450 × 10^−4^. For the case of Models 2 and 3, their correlation is more significant than 0.9. Model 1 presented difficulties in determining the content of maturity stages turning to red. This is reflected in the increase in its average error, as shown in Figure 2. 

Figure 2 shows the average error for Model 3 and Model 6. They are very near to zero. Together, it can be seen that Model 6 presents the most significant error of 6.29%.

**Figure 3:**
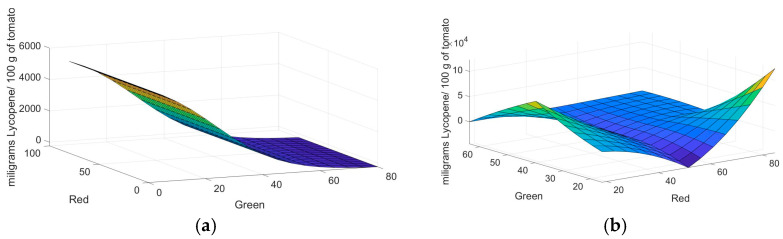
Resulting surface from the causality of the red and green inputs in the proposed models. (**a**) Model based on ANNs using sigmoidal-type activation functions. (**b**) Model based on FL using triangular-type membership functions.

In order, Figure 3 shows the response of the proposed Model 1 and Model 3. Both models can observe an increase in the lycopene content of the samples when the red pixels increase and the green pixels decrease. The increase in lycopene is related to the change in the tomato’s maturity. These results coincide with the visual classification of the samples by their degree of maturity and present a good correlation with the measurements made by the proposed sensor and the high-performance liquid chromatography (HPLC).

## 3. Discussion

The main contribution of this work is the design, construction, and testing of a sensor to measure lycopene in tomatoes in the field without destroying them, calibrated with HPLC and with qualities such as portability, high reliability, low energy consumption, and low cost. Table 1 shows the comparison of the different models. To find the best estimate, several mathematical models were created; for example, Model 4 was the one that presented a good correlation of R^2^ = 0.99 and the lowest prediction error, which was 1.9 × 10^−5^. Table 1 shows different proposals that include Models 5 and 6 that presented a high correlation with the lycopene content using the chromatic transformation *a/*b, as well as other proposals reported in the literature. These results coincide with what was reported by [15,18]. Together, the models that used multiple nonlinear regression show a higher correlation that is consistent with that reported by [27]. This sensor analyzes most of the surface of the epicarp of the fruit, which differs from that reported by [4,15], who performed a specific local analysis. Being non-destructive, this device allows it to be used as a tomato traceability tool from cultivation, harvest, transport, storage, and sale until consumption [28].

On the other hand, the electronic components’ industrialization has lowered the products’ costs such as cameras, touch screens, and minicomputers, and such is the case of the Raspberry Pi 4 where mathematical models were implemented. In addition, the RGB video camera and touch screen were connected to finally store all the method results in a micro USB flash drive. The advent of 3D printing also allowed us to build a functional and cheap prototype, and artificial intelligence gave us the opportunity to propose a lycopene estimation model with high reliability due to its low error with respect to HPLC. This same technology lets us use standard batteries in voltage and electrical current that allow the portability of the proposed sensor.

One of the main limitations of the investigation is the spectral range of operation, which is from 400 to 700 nm. Another limiting factor of the research is that the sensor must capture images of the samples under controlled lighting conditions. To develop a full picture of the measurement of Lycopene, we will need to increase the number of samples analyzed using HPLC. Further studies will need to be undertaken firmness, sugars, acidity, and tomato defects.

## 4. Materials and Methods

### 4.1. Samples

**Figure 4:**
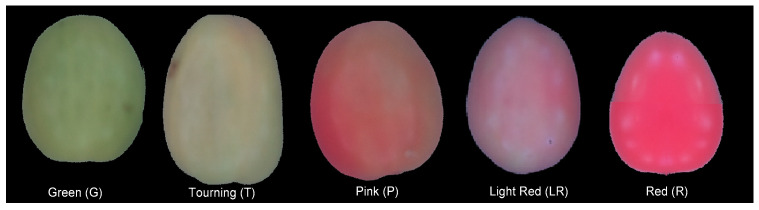
Tomato samples with different degrees of maturity.

**Figure 5:**
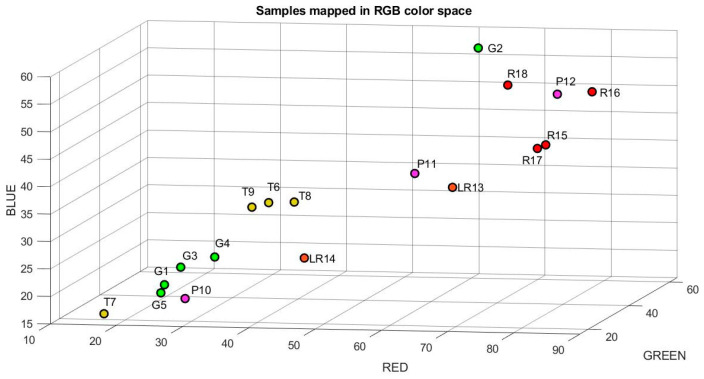
Mapping of the tomato samples using the RGB color space model.

**Figure 6:**
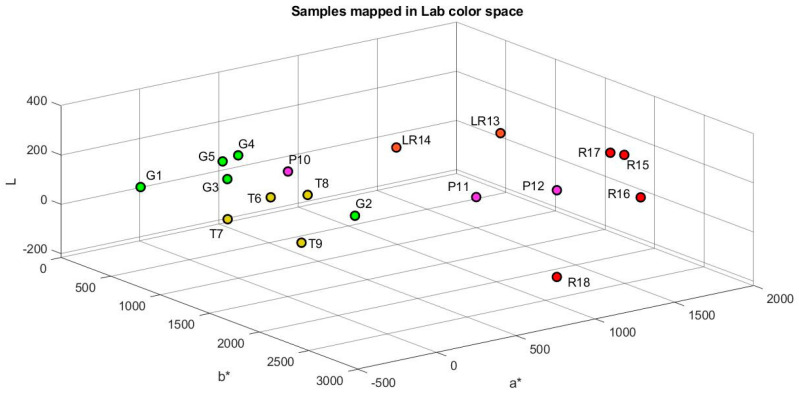
Mapping of the tomato samples using the CIE-L*a*b* color space model.

The characteristics and the number of samples were selected according to the methodology proposed by [16]. A total of 18 tomato samples that were produced in the Laja-Bajió region, Guanajuato, Mexico, were analyzed. These samples were selected with different degrees of maturity and homogeneous sizes. Tomatoes were visually classified into five maturity groups, as shown in Figure 4. These were composed of five green samples (G), three turned (T), three pink (P), two light red (LR), and five red (R). Figure 5 and Figure 6 show the mapping of the samples using the RGB and lab color models. Consequently, it is observed that there is a relation between maturity and orthogonal axes.

### 4.2. HPLC Analysis

**Figure 7:**
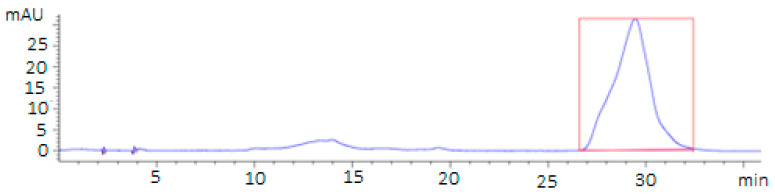
Lycopene measurement chromatogram.

The extraction of lycopene from the tomatoes was based on the methodology proposed by [15,29]. The samples were washed with distilled water and dried carefully. The tomatoes were homogenized for 5 min with a sample blender. Carotenoid extraction was carried out using 5 g of each homogenized sample. Subsequently, they were added with a mixture of solvents (hexane/ethanol/acetone 50:25:25). It was immediately stirred for 5 min. Then, it was centrifuged for 5 min, and the supernatant was extracted. This was filtered through a membrane at 0.45 µm. All solvents used were HPLC grade. Lycopene quantification was performed using an Agilent 1100 HPLC. This used a ZORBAX Eclipse XDB-C18 column (Agilent Technologies, Santa Clara, CA, USA) (4.6 × 150 mm, 5 µm) that operated with an isocratic mobile phase with a flow rate of 1.2 mL/min of methanol and acetonitrile in a 4:6 ratio. The column temperature was 30 °C, and the absorbance was read at 475 nm. The calibration curve was obtained using a 95% pure lycopene standard (Sigma Chemical Co., St. Louis, MO, USA) and showed a retention time of 31 min. Figure 7 shows the chromatogram with the retention time (minutes). The retention peak area was 4457.5 mAU (milli-absorbance unit), corresponding to 89.285 ppm.

### 4.3. Sensor Developed

**Figure 8:**
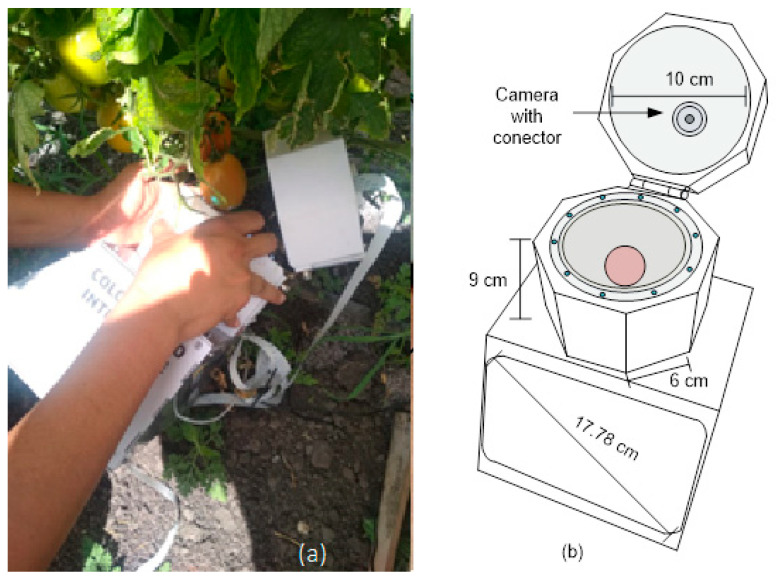
Proposed sensor for measuring lycopene in tomato: (**a**) Operation of the proposed sensor in the field, (**b**) Physical diagram of sensor.

Figure 8a shows the sensor developed for measuring lycopene. This uses an 8 Megapixel Raspberry Pi 2 camera (Raspberry Pi Foundation, Cambridge, UK) that captures the reflection of light on the tomato. This light intensity is converted into a monochrome RGB image [29]. The processing of the RGB images was carried out by the Raspberry Pi 4 development board (Raspberry Pi Foundation) and an algorithm developed in OpenCV-Python 3. The sensor has a 17.78 cm or 7-inch touch screen that is an interface that shows the captured image and the lycopene content. This works with a 10,000 mAh 9 V/5 V 2 A power bank. Together, this has a semi-spherical semi-dome with a diameter of 10 cm that maintains diffuse illumination over the sample, as shown in Figure 8b [30]. It was built with an acrylonitrile butadiene styrene (ABS) filament that has good strength and low weight. In addition, this semi-dome has a camera and connector to place filters from the MIDOPT manufacturer. The lighting has a ring-type architecture with eight white LEDs located at the same separation. This was selected for its ability to reduce the saturation of pixels in the capture of images by the camera. In addition, this sensor has an isolation system that is made up of a cylinder with a radius of 10 cm and a height that prevents disturbances from exterior lighting. This is the same material as the semi-spherical semi-dome. In addition, the architecture of the sensor can capture images using seven bandpass filters corresponding to the wavelengths of cyan, light green, orange, light red, dark red, and near-infrared/ultraviolet block-visible colors.

#### 4.3.1. Sensor Device Configuration

**Figure 9:**
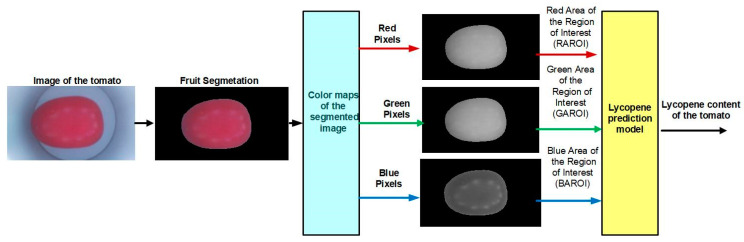
Scheme of the algorithm for the lycopene prediction in tomato.

Figure 9 shows the general scheme of the image processing algorithm proposed to predict lycopene content in tomato. This was integrated into four stages: the image acquisition, the image segmentation, obtaining the regions of interest to calculate areas, and prediction model evaluation. 

#### 4.3.2. Image Acquisition

**Figure 10:**
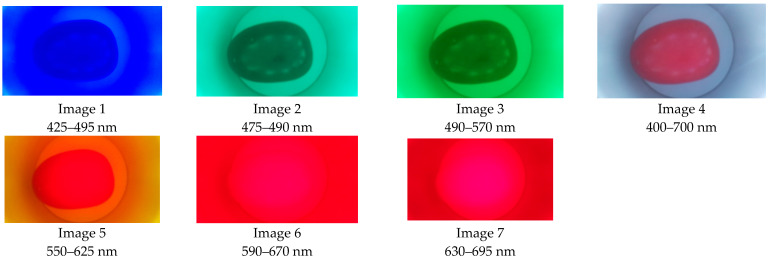
Images acquired by the sensor.

Seven RGB images were taken for each sample using seven filters, as shown in Figure 10. The image format was “JPG” with a resolution of 768 × 1366 × 3 [31]. The illumination spectrum is in the range of 400 to 800 nm [32]. This lighting affects the semi-dome in order to have diffuse lighting on the sample [33].

#### 4.3.3. Fruit Segmentation

**Figure 11:**
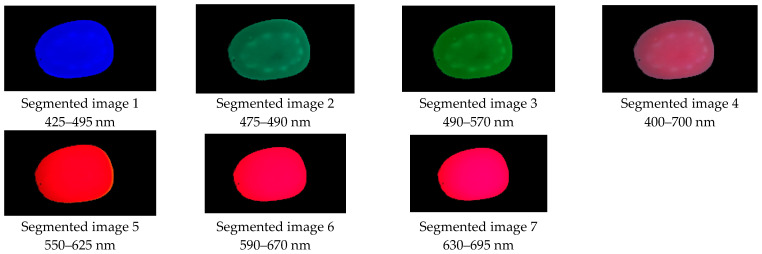
Images segmented by the sensor.

The segmentation of the background of the sample was carried out using the HSV color space model. This has the advantage that the frequencies of each color in the visible spectrum can be identified. The H component is associated with the color hue, the purity of the color with the S component, and the proximity of the pixel to black and white with the V component. The segmentation was carried out with a range of H from 0.842 to 0.466, and for the case of S and V it was from 0 to 1. Figure 11 shows the results of the segmentation of the six views. Subsequently, the areas of pixels smaller than 400 that were not part of the samples were discriminated.

### 4.4. Lycopene Estimator

#### 4.4.1. Artificial Neural Networks (ANNs)

Artificial neural networks (ANNs) are computational models that emulate the nervous system. These have become important due to their application in complex tasks of classification of maturity, shapes, and fruit defects. The ANNs are integrated by a set of neurons that are also known as nodes. The nodes can be classified as input, hidden, and output. The input nodes are stimulated by the information they receive from the outside. The hidden nodes are in charge of transmitting the information between the nodes of the network. The exit nodes send the processed information over the network [34,35].

**Figure 12:**
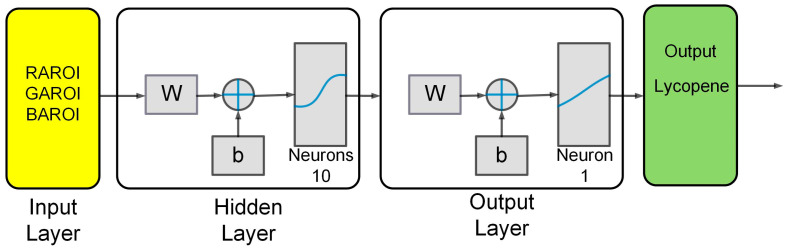
Artificial neuronal network predictor of lycopene.

Figure 12 shows the ANN architecture proposed to estimate the lycopene content. The proposed architecture has three layers. The first layer is the input, in which the training and validation data set corresponding to the color present in the tomato epicarp is presented. These correspond to the areas of the regions of interest corresponding to the red, green, and blue pixels. The input layer used sigmoidal-type stimulated functions that are activated by the training pattern. The hidden layer adds the values of each neuron from the input layer that are multiplied. If the weight value is a positive number, it stimulates the next neuron. If the weight value is a negative number, then the next neuron will not be stimulated. The output layer is linear.

Table 2 shows the characteristics of the proposed ANN models. The training of the models was carried out with the MATLAB Neuronal Network Toolbox. Model 1 used the regions of the RGB pixels, 10 neurons in the hidden layer of the sigmoidal type, and a linear output neuron as inputs. This achieved a correlation of R^2^ = 0.98 and an average error of 0.1684. The difference in Model 2 was the use of inputs in the L*, a* and b* pixel regions. This achieved a correlation of R^2^ = 0.90 and an average error of 0.5084. The proposed models are trained with the Levenberg–Marquardt algorithm. It has the advantages of a fast convergence mean square error (MSE) and an ability to approximate functions despite their complexity.

#### 4.4.2. Fuzzy Logic (FL)

Fuzzy Logic (FL) is an artificial intelligence technique that allows you to analyze vague and imprecise data efficiently. This is based on the theory of fuzzy sets, which has the characteristics of utilizing an infinite number of truth values and linguist variables.

**Figure 13:**
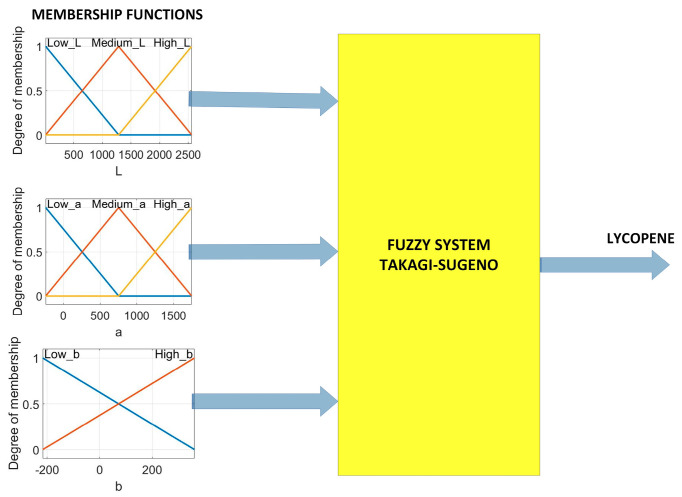
Fuzzy predictor of lycopene.

The main elements that integrate into a fuzzy system are fuzzification, inference, and defuzzification. Figure 13 shows the architecture of the fuzzy prediction system proposed for lycopene. The architecture has four inputs, one output, eight memberships, and eighteen rules of inference. 

Fuzzification involves the transformation of real input variables into fuzzy values. This transformation aims to determine the degree of membership of the input. Triangular functions were applied to merge the variables, selecting them due to their easy implementation on the Raspberry Pi 3. The linguistic variables used were low, medium, and high. The mathematical equations corresponding to each membership function are detailed in Equations (1)–(8).

(1) $$Low\_L=\left\{\begin{array}{c} \frac{1285-L}{1285}\;\;\;0<L\leq 1285\\ \;\;\;\;\;\;\;\;\;\;\;\;\;0\;\;\;\;\;\;1285<L\leq 2558.3 \end{array}\right.$$

(2) $$Medium\_L=\left\{\begin{array}{c} \frac{L-10.7}{1273}\;\;\;\;\;\;\;\;\;\;\;\;\;\;\;\;\;\;0<L\leq 1285\\ \;\;\;\;\;\;\;\frac{L-1285}{1273.7}\;\;\;\;\;\;1285<L\leq 2558.3 \end{array}\right.$$

(3) $$High\_L=\left\{\begin{array}{c} 0\;\;\;\;\;\;\;\;\;\;\;\;\;\;\;\;0<L\leq 1285\\ \;\;\;\;\;\;\;\frac{L-1273}{1273}\;\;\;\;\;\;1285<L\leq 2558.3 \end{array}\right.$$

(4) $$Low\_a=\left\{\begin{array}{c} \frac{753-a}{753}\;\;\;0<a\leq 753\\ \;\;\;\;\;\;\;\;\;\;\;\;\;0\;\;\;\;\;\;753<a\leq 1736 \end{array}\right.$$

(5) $$Medium\_a=\left\{\begin{array}{c} \frac{a}{753}\;\;\;\;\;\;\;0<a\leq 753\\ \;\;\;\;\;\;\;\;\;\frac{1736-a}{983}\;\;\;\;\;\;753<a\leq 1736 \end{array}\right.$$

(6) $$High\_a=\left\{\begin{array}{c} 0\;\;\;\;\;\;\;\;\;\;\;\;\;\;\;\;\;\;0<a\leq 753\\ \;\;\;\;\frac{a-753}{983}\;\;\;\;\;\;753<a\leq 1736 \end{array}\right.$$

(7) $$Low\_b=\left\{\begin{array}{c} \frac{358-b}{148}\;\;\;\;-210<b\leq 358 \end{array}\right.$$

(8) $$High\_b=\left\{\begin{array}{c} \frac{b-210}{148}\;\;\;\;-210<b\leq 358 \end{array}\right.$$

**Figure 14:**
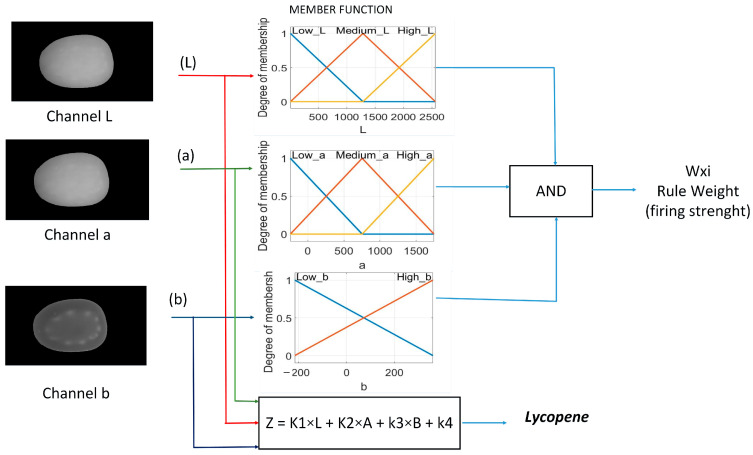
Operation of Takagi–Sugeno rules to estimate lycopene in tomato. The red line represents the luminance used in the fuzzification of the input. Also, it is used by the output level of the rule. The green and blue lines represent a and b chromaticity, respectively.

Figure 14 represents the Takagi–Sugeno fuzzy model used for the development of the classifier. The reason for this choice was the ability of the ANFIS method to quickly train fuzzy systems with this architecture. Eighteen inference rules obtained from the fuzzy values of the regions of interest were used. The transformation of the classification output fuzzy values was performed using Equation (9). The final output is determined by rules using the *Z_i_* output levels and the weight *w_i_* of the rule. The method was chosen because it uses fuzzy rules and provides a series of linear functions as output. This model allows it to analyze complex systems with larger dimensions than if the Mamdani method was used. All inference rules are found in Appendix A.

(9) $$Final\;output=\frac{\sum_{i}^{N}w_{i}Z_{i}}{\sum_{i}^{18}w_{i}}$$

Table 3 shows the models that were evaluated in the sensor using FL. Model 1 was a linear regression (LR) using a*/b* transformed chromaticity. Models 2 and 3 were multiple nonlinear neuronal regressions (MNNR) using 10 neurons of the sigmoid type. Its inputs are the RGB and CIE-L*a*b color space components, respectively. For the case of Models 4 to 6, multiple nonlinear fuzzy regression (MNFR) was used. These models used two membership functions of the triangular type for each entry. The training of the models was carried out with the MATLAB Fuzzy Logic Designer.

## 5. Conclusions

In this work, a low-cost sensor for lycopene measurement in tomatoes was developed. High reliability was found according to the results obtained with Models 4, 5, and 6, which used fuzzy logic due to the low error with respect to the HPLC measurements. The results were also compared with recent works in the literature that validate our proposal. Qualitatively, it was observed that the proposed models are sensitive to lycopene measurements at different stages of maturity. This supports the premise of measuring the lycopene content using the coloration present in the epicarp. 

Regarding costs, the proposal is based on highly industrialized and standardized components, which helps to reduce the component’s price. The cost of the sensor is around USD $100–120. It offers the advantage of operating in a time reduced to seconds to estimate the lycopene content in tomatoes, which also reduces the cost of measurement. Moreover, the fact that it is a non-destructive measurement allows it to be a helpful sensor for the traceability of the tomato from the crop to the consumption. It should be highlighted that this sensor can be used to measure other variables regarding the quality of the fruits, such as firmness, sugars, acidity, and tomato damage. In particular, fruit carotenoids such as lutein, beta-carotene, beta-cryptoxanthin, and capsanthin can be estimated using the proposed method.

## Figures and Tables

**Table 1:** Comparison of the different models.

Models	Technique	Input	R^2^	Error Mean
Model 1	MNNR	R, G, B	0.98	0.1684
Model 2	MNNR	L*, *a, *b	0.90	0.5084
Model 3	MNFR	R, G, B	0.99	−9.53 × 10^−7^
Model 4	MNFR	L*, *a, *b	0.99	0.9006
Model 5	MNFR	*a/*b.	0.99	0.0286
Model 6	MNFR	R, G	0.99	0.9006
(Arias et al., 2000) [15]	LR	L*, *a, *b	0.90	6.2911
(Vazquez-Cruz et al., 2013) [17]	MNNR	L*, *a, *b, LAI	0.95	3.75 × 10^−5^
(Tilahun et al., 2018) [18]	LR	*a, *a/*b	0.92, 0.94	
(Goisser et al., 2020) [28]	Exponential regression	L*, a*, b*, TCI	0.94, 0.90, 0.90, 0.91	

**Table 2:** Proposed models for measuring lycopene in tomatoes using ANNs.

Models	Technique	Entry	Neurons in the Hidden Layer Sigmoid	R^2^	Error Mean	Epochs
Model 1	ANNs	R, G, B	10	0.9865	0.1684	10
Model 2	ANNs	L*, a*, b*	10	0.9997	0.5084	10

**Table 3:** Proposed models for measuring lycopene in tomato using FL.

Models	Technique	Entry	Neurons in the Hidden Layer Sigmoid	Triangular Membership Features	R^2^	Error Mean	Epochs
Model 3	FL	R, G, B	-	8	0.9900	1.9 × 10^−5^	10
Model 4	FL	L, *a, *b	-	8	0.9900	−0.9006	10
Model 5	FL	*a/*b	-	8	0.2896	−7.8 × 10^4^	10
Model 6	FL	R, G	-	8	0.9900	−1.336 × 10^−6^	10

## Data Availability

Data supporting reported results can be found at: https://drive.google.com/drive/folders/1d1Q_RtEWmo2lbpipMCNG4x53s09-pB-C?usp=sharing.

## Appendix A

Listed below are the 18 inference rules and weights for each of the two fuzzy systems red, green, and blue:

1. If (L is Low_L) and (a is Low_a) and (b is Low_b) then (Lycopene is Lycopenemf1)
2. If (L is Low_L) and (a is Low_a) and (b is High_b) then (Lycopene is Lycopenemf2)
3. If (L is Low_L) and (a is Medium_a) and (b is Low_b) then (Lycopene is Lycopenemf3)
4. If (L is Low_L) and (a is Medium_a) and (b is High_b) then (Lycopene is Lycopenemf4)
5. If (L is Low_L) and (a is High_a) and (b is Low_b) then (Lycopene is Lycopenemf5)
6. If (L is Low_L) and (a is High_a) and (b is High_b) then (Lycopene is Lycopenemf6)
7. If (L is Medium_L) and (a is Low_a) and (b is Low_b) then (Lycopene is Lycopenemf7)
8. If (L is Medium_L) and (a is Low_a) and (b is High_b) then (Lycopene is Lycopenemf8)
9. If (L is Medium_L) and (a is Medium_a) and (b is Low_b) then (Lycopene is Lycopenemf9)
10. If (L is Medium_L) and (a is Medium_a) and (b is High_b) then (Lycopene is Lycopenemf10)
11. If (L is Medium_L) and (a is High_a) and (b is Low_b) then (Lycopene is Lycopenemf11)
12. If (L is Medium_L) and (a is High_a) and (b is High_b) then (Lycopene is Lycopenemf12)
13. If (L is High_L) and (a is Low_a) and (b is Low_b) then (Lycopene is Lycopenemf13)
14. If (L is High_L) and (a is Low_a) and (b is High_b) then (Lycopene is Lycopenemf14)
15. If (L is High_L) and (a is Medium_a) and (b is Low_b) then (Lycopene is Lycopenemf15)
16. If (L is High_L) and (a is Medium_a) and (b is High_b) then (Lycopene is Lycopenemf16)
17. If (L is High_L) and (a is High_a) and (b is Low_b) then (Lycopene is Lycopenemf17)
18. If (L is High_L) and (a is High_a) and (b is High_b) then (Lycopene is Lycopenemf18)

The coefficients obtained by the corresponding ANFIS are the following:

W1 = [−6.69138547328559 −1.11067084181724 4.34025725184568 0.93184936978667]

W2 = [−0.318478847326583 5.64788126870901 3.59437092828302 0.58175498089429]

W3 = [ 3.95014047840935 1.59052198265991 3.3900952788002 0.299852425910745]

W4 = [19.5035468323403 6.38471348304066 4.78233606536508 0.202776907148019]

W5 = [0 0 0 0]

W6 = [0 0 0 0]

W7 = [3.16961759692625 2.58260252215251 −7.47834846471906 −0.0159918054557269]

W8 = [−2.49662488559166 −12.3987736560711 1.58390104162457 −0.0259522172477145]

W9 = [−0.963673706231771 −7.67628285198772 −14.882070217473 −0.000978530340102667]

W10 = [−1.17652671474686 4.28133259162214 8.58933040834423 −0.0180386146926471]

W11 = [27.6837746525357 30.0998637574663 −6.04580283260799 0.0125205680628005]

W12 = [−12.2501695143382 −9.11572464548948 1.28641408118073 −0.00567972816519314]

W13 = [3.89196417128968 −5.62446094757007 0.449363113520893 0.00425330019057446]

W14 = [2.09041280770293 −14.8185805057198 1.75403548818368 0.0086653984730775]

W15 = [−3.74341236002276 −16.3575329602085 −13.1859975500766 −0.0010654550733953]

W16 = [25.7278702631458 −27.0060723666553 22.5846060837755 0.0254252141584876]

W17 = [−17.3655978345049 16.2129055618654 −5.54378482385434 −0.00225230673875521]

W18 = [−24.9130829636862 38.4699087639502 1.82213892573319 −0.0116749561770817]
